# Religion, Culture and Meaning-Making Coping: A Study Among Cancer Patients in Turkey

**DOI:** 10.1007/s10943-018-0646-7

**Published:** 2018-06-05

**Authors:** Fereshteh Ahmadi, Pelin Erbil, Nader Ahmadi, Önver A. Cetrez

**Affiliations:** 1 Department of Social Work and Psychology, Faculty of Health and Occupational Studies, University of Gävle, 801 76 Gävle, Sweden; 2 Clinic of Humanite Psychiatry, Istanbul, Turkey; 3 Faculty of Theology, Uppsala University, Box 511, 751 20 Uppsala, Sweden

**Keywords:** Cancer, Coping, Meaning-making coping, Religion, Culture, Turkey

## Abstract

The present paper looks at the influence of culture on Turkish cancer patients’ use of meaning-making coping, paying particular attention to religious, spiritual, and existential coping methods. Data were collected using an interview study (*n* = 25, 18 women, age range 20–71). Individuals were recruited at an oncology center and a psychiatry clinic in Istanbul. The main focus of the study has been on existential meaning-making coping, which is characterized by finding power inside oneself, altruism, family love, a search for meaning by contemplating philosophical issues, and having a positive life perspective (*shukran*—thankfulness). In contrast to findings from similar studies conducted in other countries (studies included in the same project), in Turkey religious belief directly determines the coping methods used, including the non-religious methods.

## Introduction

To look more closely at the role of culture in cancer patients’ use of meaning-making coping, an international project has been conducted in five countries (Sweden, South Korea, China, Japan, and Turkey). The purpose of the project has been to focus on different forms of meaning-making coping (existential, spiritual, and religious coping) among people diagnosed with cancer and to try to understand the influence of culture on the use of these coping methods. In the present study, we refer to culture as a system of beliefs, traditions, customs, art, history, folklore, institutions, norm and values and their explicit expression as shared by members of a society, a community, or a group.

Our specific questions were:

- Which of the religious and spiritually oriented coping methods used by cancer patients can be categorized as religious coping, as defined by the Many Religious Coping Methods (RCOPE)?
- What has the role of culture been in the choice of religious and spiritually oriented coping methods?

In the current article, which is based on a study carried out in Turkey, we present results on religious, spiritual, and existential meaning-making methods of coping with cancer. A detailed discussion on the religious coping methods has already been presented in another paper (Ahmadi et al. 2016a). Here, these methods will be discussed briefly.

In the present study, the term meaning-making coping is used to refer to the entire spectrum of religious, spiritual, and existential coping methods (ibid.).

## Methodology

Interview questions for the study in Turkey were mainly constructed based on the results from the Swedish study (Ahmadi 2015). Two psychologists translated the original interview guide from English to Turkish and back to English. Some terms were modified to better suit the Turkish culture; for instance, “church” was replaced by “mosque” and “God” by “Tanrı.” In daily conversations among older generations and in religious environments, the word “Allah” is more commonly used, but for the new generations the word “Allah” has a religious connotation; thus, in the interviews the word “Tanrı” (deity) was used.

For the present qualitative study, we chose a convenience sample. A total of 25 participants (18 women and 7 men) were recruited in Istanbul. Psychologists at an oncology center and a psychiatry clinic informed their patients about the study. These patients had either survived cancer or were still undergoing chemotherapy and/or radiotherapy. Upon receiving the patients’ consent to participate in the study, the interviews were conducted.

### Procedure

Prior to each interview, the participants read and signed an informed consent form explaining the study purpose and ensuring confidentiality. Face-to-face interviews in Turkish were conducted at the hospital or the clinic, by the same interviewer; they lasted from 40 to 60 min, and a semi-structured questionnaire was used. The interviews were conducted from April 2015 through July 2015; the transcriptions were completed by the end of November 2015. The interviews were transcribed verbatim by the same interviewers to avoid divergence in the interpretations. Once all the interviews had been completed, the same person translated them to English.

The group of participants included both men and women, between 20 and 71 years of age. Among the 25 participating patients, the number of women far exceeded the number of men (18 and 7, respectively). They identified themselves as non-religious, spiritual, or religious. Two people reported not having a religion, though this does not mean they deprecated spiritual or existential coping. The participants had various types of cancer. Their stage of cancer varied from earliest to severe; their social status also varied, including housewives, retirees, and the currently employed. Most participants were living with their spouse and had children; 5 were 20–39 years of age, 14 were 40–59 years, and 6 were 60 + . Ten participants were high school graduates or less, and 15 college graduates or higher. Concerning employment status, 1 participant was a housewife, 11 were not working (because of the diagnosis and the treatment schedule, patients stopped working for undetermined period), 10 worked, and 3 were retired or on leave. With regard to their religious affiliation, 21 were Muslims, 2 believed in God but did not have any religious affiliation, and 2 were atheists.

### Method of Analysis

Following transcription, the interview data were coded based on the themes found in the study using a template analysis style, a theory-driven analysis (Malterud 2014). Categorization of the themes was based on a modified version of Pargament’s RCOPE used by Ahmadi in the qualitative Swedish study (Ahmadi 2006: 45–47) as well as the results of both the qualitative and quantitative Swedish studies (Ahmadi 2006, 2015). The three themes—religious, spiritual, and existential coping—were each divided into categories, such as finding meaning, gaining control, gaining comfort, among others (see “Appendix 1”). These categories, in turn, were specified by sub-categories, such as benevolent religious reappraisal, spiritual prayer, and search for meaning. The categories or sub-categories were then linked to relevant codes in the material, such as the code “Wondered whether God had abandoned me” for the category “Punishing God reappraisal,” or the code “Illness caused me to appreciate life” for the category “Benevolent religious reappraisal.” The coding continued until the inter-rater agreement reached a high level. After the coding process, we established the essential characteristics of the different methods the informants used to cope with cancer. Here we proceeded from the project aim, using the results of previous studies in the project (Ahmadi 2006, 2015). Regarding religious coping, we proceeded from the Five Key Religious Functions that constitute the basis of RCOPE (Pargament et al. 2000: 521).

### Ethical Considerations

Within the framework of the present study, we gathered data on the meaning-making coping strategies used by people who are very vulnerable given that they are facing life-threatening disease. It is clear that bringing these issues to the fore, which is related to very fundamental layers of an individual’s ego, is of ethical relevance.

This being the case, it is essential to establish a cooperative relationship with potential respondents—one that is based on trust. Here, we made it clear to potential respondents that: participation was voluntary; data would be treated with confidentiality and not made available to anyone outside the research team; agreement to participate could be canceled at any time without any consequences for the respondent; and the results obtained from the study would be published such that identification of individual respondents would be impossible.

This study was approved by the Regional Ethics Board in Uppsala (Reg. no. 2015-126).

Below, we provide a concise list and brief discussion of the meaning-making coping methods the Turkish participants reported using during their illness.

## List of Meaning-Making Methods Found in the Turkish Study

Proceeding from our definition of religion, spirituality, and existential meaning-making coping and based on previous studies in Sweden, South Korea, and China, we have observed the use of several meaning-making coping methods among our interviewees in Turkey (see “Appendix 1”).

### Religious and Spiritual Coping Methods

The present results show that the RCOPE methods seem to be highly relevant for the Turkish interviewees. This can be explained by the fact that, for these interviewees, religion is a “larger part of [their] orientation system” (Ahmadi 2006: 28). In another paper on the same study (Ahmadi et al. 2016a), we discuss the use of these methods in detail. In the present article, we provide a brief summary of some of the most important religious coping (RCOPE) methods used by interviewees in the Turkish study.

#### Benevolent Religious Reappraisal

Using the coping method benevolent religious reappraisal, the individual tries to find a lesson from God in the event or to see how the situation could be spiritually beneficial. In the present study, we found two patterns. In the first pattern, we can see the act of talking with God, prayer itself, as a way of dealing with the stressor and feeling relaxed. In the second pattern, praying seems to have helped the interviewees reexamine their past life and find a lesson there (Ahmadi et al. 2016a).

#### Passive Religious Deferral

This method involves passively waiting for God to control the situation. Regarding illness as a test that God imposes on us is an old idea. In our study (Ahmadi et al. 2016a), we found interviewees who used this method, and who explained it by referring to the idea of *Sabr* (patience).

Passively facing one’s illness and totally relying on God is, according to some researchers (Aflakseir & Coleman 2011: 46), common among Turkish people. The idea of being tested (*Ekhtebar*) and being patient (*Sabr*), i.e., the idea that the problems of this world are intended to test people and encourage them to have patience when facing problems, is quite influential among Muslims, including those in Turkey.

#### Active Religious Surrounding

This method entails actively giving up control to God (“I did my best and now I am relying totally on God”). Among our interviewees, we saw belief in the idea of *Kader,* which means “fate” or “predestination” (Ahmadi et al. 2016a). *Kader* is the concept of divine destiny found in Islamic thought. According to some Muslims, God wrote down in the *Preserved Table* (“al-Lauḥ al-Maḥfūẓ”) all that has happened and will happen. This means that God has dictated everything that happens in the world (cosmos) on the basis of his prior knowledge and the states of his wisdom. Here we are not dealing with interviewees who passively accept their destiny. Instead, they actually do their best, but nonetheless know that, ultimately, everything is in God’s hands.

#### Pleading for Direct Intercession

This method refers to seeking control indirectly by pleading to God (praying). The study indicates that some interviewees tried to gain comfort by pleading to God to make things turn out positively (Ahmadi et al. 2016a). As the study shows (ibid.), the pleading method has had a positive effect on the interviewees. It is not only pleading that plays a positive role in gaining comfort, but also their strong faith. The risk of becoming depressed or thinking one has been abandoned by God does not seem to be great among the interviewees who used this method.

#### Spiritual Discontent

This method involves expressing confusion and dissatisfaction with God’s relationship to the individual in the stressful situation (“God has abandoned me”).

By questioning the existence of God, some interviewees showed their discontent with religion and faith. The problem these interviewees faced is called the *theodicy problem.* They wonder why God, who is supposed to love his children, would expose them to such a terrible illness.

Besides these religious and spiritual methods, we also found several existential meaning-making coping methods. In the following, we will discuss the methods of existential meaning-making coping we found most interesting.

## Existential Meaning-Making Coping

### Finding Power Inside Oneself

Some interviewees have used existential meaning-making coping, finding power inside oneself, as a way to deal with the stressors brought about by cancer. As the same study conducted in Sweden (Ahmadi 2006) also confirmed, finding oneself and searching for inner possibilities as well as actualizing one’s inner force may be a spiritual outcome. As Ahmadi (2006: 112) explains, a spiritually oriented person who faces difficult events may search for the “sacred” in herself/himself and find a force greater than the self—a source of strength. Ahmadi emphasizes that “Here, we are neither witnessing ‘the incorporation of the sacred into the self’” (Pargament 1997: 253), as is the case in religious conversion, nor ‘dying unto oneself’ or ‘loss of self’ through unification with a greater existence, as in the case of self-inhibition” (Ahmadi & Ahmadi 1998). What we are perhaps witnessing is what Fromm (1950) calls *self*-*realization* when he discusses “humanistic religion.” Through realization of the self when facing a stressful situation caused by illness, some of our informants found a way to give meaning to their life and, thereby, cope with their illness.

### Altruism (Being a Good Person)

Altruism, expressed by informants as “being a good person,” was found to be one of the existential meaning-making coping methods used by some informants.

Ahmadi (2006: 139) explains:

“Human altruism is not only an act, but is interwoven with an emotion: empathy. Altruism is then as a prosocial behaviour a voluntary intention/act to help others at some cost to oneself (time, effort or money)” (ibid.).

A similar coping method was found in the study among Swedes (Ahmadi 2006: 139–141).

Although Swedish and Turkish ideas about what constitutes prosocial behavior seem to be similar, there are disparities between individualistic versus collectivistic ways of thinking. If among Swedes altruism takes the form of improvement of *la Condition Humaine* (Ahmadi 2006: 140), in the Turkish case, it concerns helping people in need in one’s vicinity and is primarily construed as a religious duty.

Below, Hinde and Groebel (1991: 90) explain the reason for differences in prosocial behavior connected to individualistic and collectivistic ways of thinking. It should be mentioned, however, that they are referring mainly to China and Japan and that most countries in Asia are not individualistic but collectivistic.

“Asian considerations of what constitutes prosocial behavior are very similar to those of the west: altruism, kindness, considerateness, sympathy, aiding…

However, Asian considerations differs from those in the west because of their conception of the role of the individual in relation to family and society. We in the west place great emphasis on the importance of the individual and on the development of an independent, self-directed child.”

In Eastern societies, however, the emphasis is on the interrelation between the child and members of the family and society (ibid.).

Another factor that should be taken into consideration here is religion. In societies where religion plays a significant role in the interrelation between people, any sense of altruism may be related to religious belief and duties. In Islam, altruism takes the form of hospitality and generosity, which are foundational virtues. There are several duties like *fasting*,^1^*zakat*,^2^ and *sadageh*,^3^ which are intended to help develop a sense of empathy and altruism among Muslims (Homerin 2005). In Turkey, this factor, i.e., the role of religion, was quite noticeable in our interviews concerning this coping method.

### Family Love

In a collectivistic society like Turkey, the importance of family gives rise to another coping method: “Family love.”

As Mahoney et al. (2003: 222) maintain:

“For many people, family relationships involve more than biological, psychological, and social processes; people often believe these bonds tap directly into the spiritual realm…in short, people often view family relationships as sacred.”

According to these authors, people can sanctify the entire family system (ibid.).

As our study among cancer patients in Turkey shows, sanctification of the family may serve as a coping method. Actively trying to get better and not allowing the problems of illness to disturb family relations or make family members unhappy can be a potent method of dealing with the psychological problems brought about by cancer. In this connection, religion may play an important role. According to Zimmerman (1974: 6), “the most sacred or divine aspect of society is considered to be family system and being religious is tantamount to being a good husband, a good wife, or a good parent, child or kinsman.”

Given that our interviewees, cancer patients in Turkey, were socialized in a group-oriented society, where religion is an important component of the dominant culture, it is understandable that “love of family” seems to have played a role in their existential meaning-making coping.

### Search for Meaning by Contemplating Philosophical Issues

We found that, for some of our interviewees, the search for existential meaning, i.e., thinking about the meaning of life and death, has functioned as an existential meaning-making method of coping with their illness.

In the Turkish case, the interviewees were believers who were contemplating existential issues in the context of religiosity.

### Positive Life Perspective (Finding New Meaning in Life by Changing Priorities)

We found that going through the painful journey of having cancer caused some interviewees to appreciate their present life and the moments they experienced in everyday life. This brought about “a turning point” that caused them to change their proprieties in life. This new attitude has helped them cope with their illness, called “positive life perspective coping method” (Ahmadi et al. 2016b).

## Conclusion

The striking finding from our study in Turkey is that, unlike the other studies in this project (already completed studies among cancer patients in Sweden, South Korea, and China), here we found a great impact of religion on the selection of meaning-making coping methods, concerning not only the religious coping methods, but also the non-religious ones. The two following examples clearly illustrate this difference.

As explained before, in the Turkish study, the search for existential meaning, i.e., thinking about the meaning of life, was found to be an existential meaning-making method of coping with their illness.

The same study conducted in Sweden (Ahmadi 2006) also showed the use of this method by some interviewees, but there is one important difference between these two cases. In the Swedish study, all of the interviewees who had used this method did not believe in God or were not religious. Being socialized in a rational and secular culture may prevent a Swede from looking to religious texts to find answers pertaining to the meaning of life and death. Yet the tendency toward spirituality in Swedish ways of thinking means that it is also not easy to satisfy a Swede by offering a simple, materialistic answer. For some Swedes, always being a seeker may give meaning to life. It would seem that, in the Turkish case, the interviewees were believers and thinking about existential questions occurred in the context of religiosity.

Another example concerns the positive life perspective (finding new meaning in life by changing priorities). As mentioned above, in the Turkish study, we found that, for some informants, being hit by cancer was “a turning point” that caused them to begin to appreciate their life and everyday life events. This has helped them cope with their illness.

In the project, the same coping method was found among Korean informants (Ahmadi et al. 2016b). Like the Korean informants, the Turkish informants used the positive life perspective coping method. But whereas in the Korean study a strong sense of responsibility was important to the change in attitudes toward a positive life perspective, in Turkey it seemed to be more of a religious attitude, namely Shukran (thankfulness), that had given rise to this positive perspective. According to Islam, one of Muslims’ important duties is to be grateful to God for all of His blessings.^4^ When something bad happens to a person, she/he thanks God that something worse did not happen; this attitude seems to reconcile people with the situation, even if it is terrible.

Comparing our results from Turkey with findings from other countries—Ahmadi (2015); Ahmadi et al. (2016b)—we assume that in those other studies as well, the cultural features of the society in which the cancer patients were socialized have played an important role in their coping methods. It would seem that, in Turkey, religious beliefs *directly* determined the interviewees’ chosen coping methods.

As the above presentation of the non-religious coping methods used by our Turkish interviewees indicates, in all non-religious coping—i.e., finding power inside herself, altruism (being a good person), family love, search for meaning by pondering philosophical issues, and positive life perspective (finding new meaning for life by changing priorities)—the interviewees who chose these coping methods had been influenced by Islamic beliefs. These beliefs have an obvious impact on the everyday life of Turkish people, despite the fact that the state has, until recently, played an assertive role in confining religion to the private sphere (also referred to as *laïcité*).

To understand such a direct convergence of religion and coping, we should remember that, as Pargament (1997: 144) mentions, religion is more available to the individual when it plays a more important role in her/his orienting system. In this respect, Ahmadi (2006: 29) explains that:

“one reason people turn to religion in a time of crisis is that religion is more accessible in their sociocultural context than are other resources. In other words, people have more access to religion as a tool for coping with difficult situations when their religious beliefs, feelings and practices are a part of their culture, and therefore a part of their orienting system.”

Although Turkey is at the intersection of strong religiosity and secularism, according to the World Values Survey, this country, with its 99.9% Muslim population, is characterized by a highly homogenous culture, traditional values, and strong religious commitment, all of which impact the way people deal with their everyday life, including their life crises.

## Appendix 1

See Table 1.

**Table 1:** List of themes, categories, and sub-categories used for analysis

Themes	Categories	Sub-categories
1. Religious coping	1.1 Find meaning	Benevolent religious reappraisal Punishing God reappraisal
	1.2 Gain control	Passive religious deferral Active religious surrounding Pleading for direct intercession Pleading for direct intercession Collaborative religious coping
	1.3 Gain comfort and closeness to God	Religious purification Spiritual discontent
	1.4 Gain intimacy with others	Seeking support from clergy or congregation members
2. Spiritual coping	2.1 Gain control	Spiritual prayer
3. Existential coping	3.1 Finding meaning	Search for meaning Appreciating life Life experience and existential feeling Finding meaning in life by hoping
	3.2 Gain control by an inner existential connection with oneself	Positive solitude Self-directing coping
	3.3 Gain comfort and closeness to a higher entity	Nature as a coping method Visualization
	3.4 Gain intimacy with others	Family love Altruism
	3.5 Achieve a life transformation	Finding new meaning for life by changing priorities

In the original theoretical model, more categories and sub-categories are found. However, the above table only consists of categories found in the analysis in the present study

## Footnotes

1 Fasting is an act of empathy. The fast is intended to remind Muslims of the suffering of those less fortunate than themselves. Muslims often donate to charities during the month and feed the hungry.

2 Zakat, the giving of alms to the poor, is one of the five pillars of Islam.

3 Donation.

4 We read in the Quran, “Why should God make you suffer torment if you are thankful and believe in Him? God always rewards gratitude and He knows everything” (Quran, 4:147).
